# Evaluation of commercially available class A water-based foam concentrates for swine depopulation

**DOI:** 10.1371/journal.pone.0328073

**Published:** 2025-08-18

**Authors:** Dawn M. Torrisi, Magnus R. Campler, Janice Y. Park, Ting-Yu Cheng, Brad L. Youngblood, Talita P. Resende, Michael D. Cressman, Justin D. Kieffer, Andréia G. Arruda, Andrew S. Bowman

**Affiliations:** 1 Office of the Attending Veterinarian, Enterprise for Research, Innovation and Knowledge, The Ohio State University, Columbus, Ohio, United States of America; 2 Department of Veterinary Preventive Medicine, College of Veterinary Medicine, The Ohio State University, Columbus, Ohio, United States of America; 3 Department of Animal Sciences, College of Food, Agricultural, and Environmental Sciences, The Ohio State University, Columbus, Ohio, United States of America; North-Caucasus Federal University, RUSSIAN FEDERATION

## Abstract

Currently, the swine industry is lacking an efficient method for large-scale emergency depopulation. Class A water-based foam (WBF) has been demonstrated as a viable option for large-scale depopulation of pigs in all stages of development. However, these studies exclusively used the PHOS-CHEK WD881 (WD881) Class A foam concentrate based on previously demonstrated efficacy for depopulation. This study investigated the suitability of 15 other commercially available WBF concentrates for depopulation based on foaming performance, physiological effects, and efficacy. The performance of each product was evaluated and compared to WD881 at 0.5, 1.0 and 3.0% water-foam concentrations for low- and high-pressure pump systems. The time to fill an 11.5 m^3^ construction container and decay rate over a 10-minute dwell time were assessed for each WBF. Top-performing foams were further evaluated for behavior and short-term physiological changes and gross lesions during a 15-minute exposure test on piglets. Finally, the top-performing foams were tested for their suitability to depopulate adult swine during large-scale field conditions. Subcutaneous dataloggers recorded swine activity which was used to estimate the time to cessation of movement (COM), an approximate analog for loss of consciousness. Four WBF concentrates (FireIce Polar EcoFoam, Buckeye Platinum, National Foams Knockdown and BioFor N) were shortlisted based on performance at 1% concentration. These products had a mean (±SD) fill time of 62.4s (± 14.9) and decay rate of 0.5 (± 0.66) cm/min compared to WD881 with 50.0s (± 3.5) and 0.2 (± 0.1) cm/min, respectively. No differences between treatment groups were observed during the exposure testing and subsequent necropsy. For the large-scale field trials, the mean (±SE) time to COM was 151.5 s (±10.5). All foams achieved 100% mortality of swine. This study identified four additional WBFs suitable for swine depopulation which are commercially available on the U.S market. These additional WBF options may facilitate large-scale swine depopulation during widespread infectious disease outbreaks by mitigating potential bottlenecks resulting from product availability.

## Introduction

Food animal species are subject to a host of conditions that could necessitate emergency destruction of large numbers of individuals. Threats such as infectious diseases, contamination of feed or water sources, radiation exposure, or natural disasters may result in depopulation as the only viable response [1]. Due to high density and frequent movement of animals, commercial livestock and poultry producers are particularly vulnerable to emergency conditions that may necessitate rapid depopulation to prevent animal suffering, hasten inevitable mortality, and prevent the spread of disease [2,3]. However, large-scale depopulation of mammalian agricultural species, including swine, continues to be challenging. Recently, Kaiser, et al. developed a portable CO2 delivery system that can be used for large scale depopulation of swine [4]. However, some producers may not have access to the specialized materials needed to use this system. Historically, methods such as CO2 gassing, movement to slaughter, or penetrating and non-penetrating captive bolt have been used to eliminate large populations of swine in emergency situations [5,6]. These methods pose logistical challenges including resource availability, mandatory quarantine, transportation, and labor for removal and disposal. Other methods preferred by the American Veterinary Medical Association, including gunshot, penetrating captive bolt, and barbiturate overdose, can be problematic due to the requirement for specialized equipment and training, labor intensity, and lack of scalability [1,5,7]. This poses significant concerns for relay toxicosis with barbiturate use and increased incidence of depopulation distress in humans performing physical methods for depopulation [7–9]. These limitations became apparent in 2020 when many harvest operations were abruptly halted in response to the SARS-CoV-2 pandemic. Many producers throughout the industry were unprepared or unable to execute large-scale emergency depopulation of vast numbers of swine, prompting the investigation into Class A water-based foam as a depopulation agent [7,10–15].

Class A foam concentrates are typically used for forest fire suppression and aerated with compressed gas, air, or water to produce dense foam. The foam clings to and covers surfaces, effectively smothering fire. The degree of adhesion of the foam may be influenced by many factors, including bubble size, which may vary according to type of foam [16]. The US Forest Service has reported product-dependent variations in the composition and performance of WBF products when used for fire suppression because foam products behave differently depending on conditions such as water temperature, pH, or the mineral content of the water [17]. Thus, although there are many WBFs options, not all produced foam may be suitable for swine depopulation.

Previous studies have shown that WBF causes rapid hypoxia, unconsciousness, and subsequent death due to airway occlusion in poultry, swine, and adult cattle, making it an effective method for large-scale depopulation [2,11–13,15,18,19]. However, the above-mentioned WBF depopulation studies on swine have been conducted using a single Class A foam concentrate, PHOS-CHEK WD881. This product was selected because it is available from the National Veterinary Stockpile and has been commonly used for poultry depopulation. Despite the inclusion of WD881 in the NVS, sourcing the product can be challenging due to variations in regional availability leading to a demand for additional suitable alternatives during a time of emergency. Based on existing information, no other Class A foam concentrates have been tested, described and published for swine depopulation to date, leaving a major gap in knowledge regarding the potential suitability of other commercially available Class A products. Therefore, the aim of this study was to identify different brands of commercially available WBFs and assess their efficacy and suitability as depopulation agents in swine to ensure nationwide availability of water-based foam products. It was hypothesized there would be other WBF capable of performing well for swine depopulation with efficacy comparable to WD881. The objectives were to 1) assess and shortlist commercially available WBFs based on the physical properties and decay rate under high- and low-pressure, 2) evaluate short-term exposure of the shortlisted WBFs for swine aversion or potential negative physiological effects, and 3) test the efficacy of each of the short-listed WBFs in a large-scale depopulation scenario during field conditions and estimate the over-all cost by employing a custom-designed algorithm.

## Materials and methods

### Ethics and institutional oversight

The Institutional Animal Care and Use Committee at The Ohio State University approved the activities in this study under the animal protocol 2020A00000036. A penetrating captive bolt was present during all phases of animal experimentation for euthanasia, if necessary.

### Foam concentrate selection

Fifteen Class A foam concentrates, each composed of a proprietary mixture of surfactants, were obtained from commercial vendors (Table 1). Dawn^®^ P&G Professional dish soap was additionally evaluated because swine industry stakeholders had voiced interest in this product as a highly accessible alternative to WBF. The performance of the 15 WBFs and the dish soap was compared to that of WD881. PHOS-CHEK WD881 is known to have appropriate physical properties to effectively depopulate swine of different ages utilizing a high-pressure foam generating system and therefore was used as the standard product [11,12].

**Table 1:** Source company and cost of a selected number of commercially available and tested Class A foam concentrates and a commercial dish soap in the United States (July/2023).

Class A Foam Concentrate	Source Company	Cost ($USD)/gallon^a^
**PHOS-CHEK WD881**	Perimeter Solutions	34.8
**Ansul Silv-ex Plus**	Ansul	20.8
**BioFor N**	BioEx	20.6
**Buckeye Platinum**	Buckeye	37.4
**Chemattack Class A Foam**	Chemguard	20.0
**Chemguard Class A Plus+**	Chemguard	20.0
**Crestar**	Crestar Fire	17.8
**Enforcer Firebull**	A.J. Stone Company, Ltd.	14.9
**FireAde**	FireAde	24.8
**FireStopper AB 40002 FFC** ^b^	FireStopper, USA	72.3
**Fire-Trol 103** ^b^	Chemtronics	24.8
**Knockdown**	National Foams	20.5
**Pinnacle**	Hazard Control Tech	23.0
**Phos-Chek First Response**	Perimeter Solutions	27.3
**Polar Ecofoam**	FireIce	27.1
**Solberg Fire-Brake**	Perimeter Solutions	19.1
**Non-Class A Foam Concentrate**		
**Dawn**^**®**^ **dish soap**	Procter & Gamble	25.0

The tested products are composed of a proprietary mixture of surfactants. Most contain chemical compounds such as sulfonates, sulfates, alcohol, ethers, and hydrocarbons [20–36].

^a^shipping costs are not included and may vary geographically

^b^foams no longer in production at the time of publication

### Foam concentrate dilution

**Fig 1:**
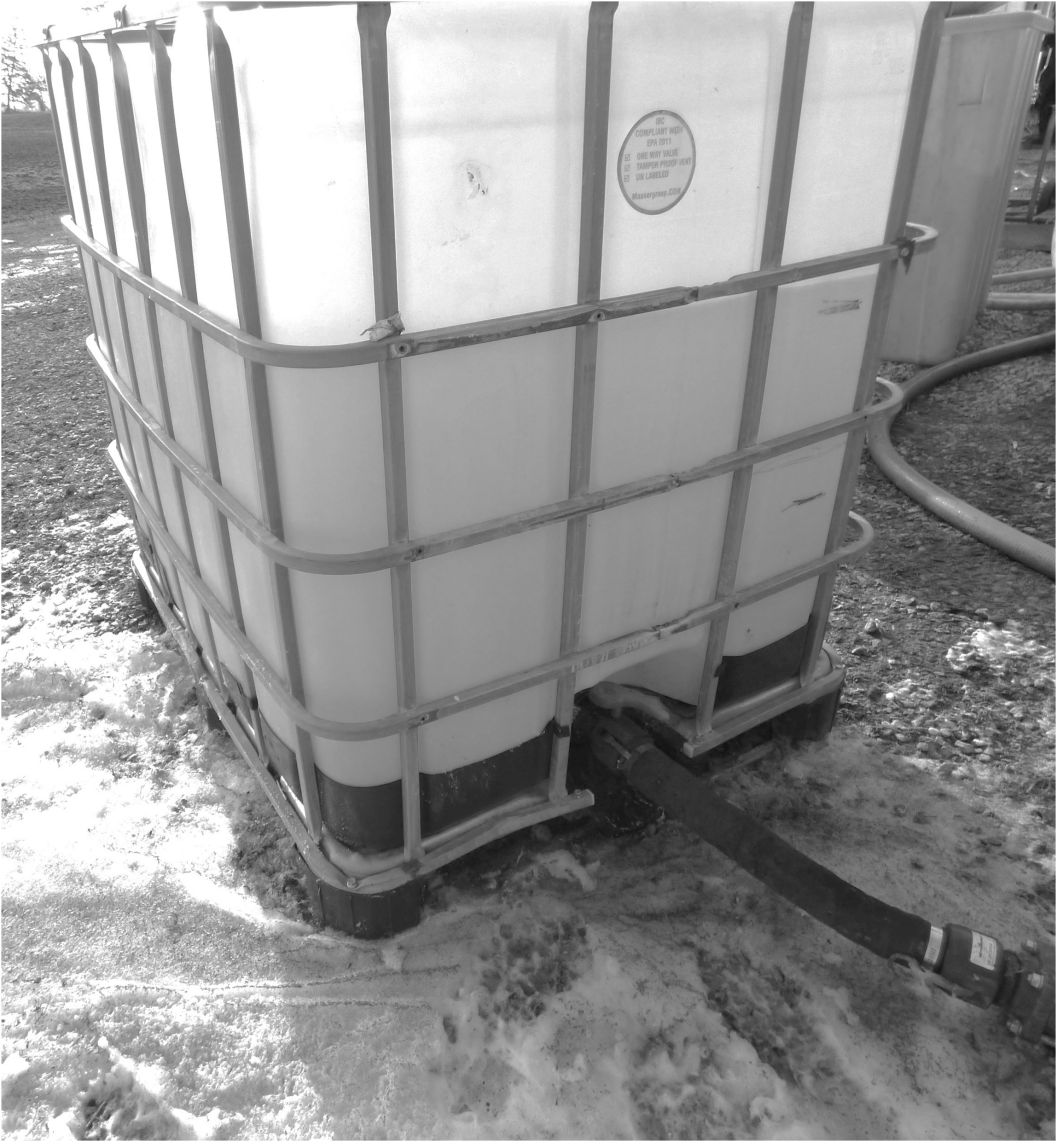
Intermediate bulk container (1136 L) with bottom drain used to mix foam-water solutions at prescribed concentrations. Fluid was pumped directly from container during foam production.

Commercial-grade intermediate bulk container tanks (available from various manufacturers) were used to mix the solutions used for producing foam. The tanks were palletized and contained within an aluminum frame. Each tank held 1249 L of fluid and had a single male cam and groove drain port located near the bottom of the tank (Fig 1). The tanks were filled with 1136 L of fresh well water through a fill port on the top of the tank. The total volume was estimated by referencing the manufacturer’s markings for volume on the side of the IBC tank.

To obtain 0.5, 1, and 3% solutions, foam concentrates were measured with a 3.79 L measuring cup and poured into the tank after filling with water to avoid production of foam as water was added. To ensure the dilutions were well-mixed, the solution was stirred for at least one minute before foam production.

### Foam production

For the low-pressure foam production, a gas-powered trash pump with 5.1 cm hose ports (BravePro, BRP500TP2, Honda, Tokyo, Japan) and a medium-expansion foam handline nozzle (AWG, Model M2 16712−3, AWG Fittings GmbH, Ballendorf, Germany) was used. For the high-pressure foam production, a gas-powered water pump (AMT Pump Company 2MP13HR, Royersford, PA, USA) with 3.81 cm hose ports and a medium-expansion foam handline nozzle (KR-M4, ANSUL, Marinette, WI, USA) was used. For both pumps, a 30.5 m rigid suction hose was connected between the drain port of the IBC and the intake port of the water pump using respectively sized (3.81 or 5.1 cm) cam and groove connectors. Similar adaptors were used to connect a 15.3 m (3.81 cm diameter) fire hose to the water pump outlet and then fitted to the medium expansion handline nozzle.

For the low- and high-pressure foam and exposure trial, foam was generated utilizing a single pump system as described. However, for the field trials, three complete high-pressure pump systems were assembled and used simultaneously to compensate for the larger fill volume to operate at the same fill rate. To maximize productivity, each set of pumps were modified to accommodate two IBCs. A female cam and groove Y-connector was utilized to connect the two IBCs to a single water pump. A manual shut-off valve was installed between each IBC and its respective suction hose, allowing for preparation of dilutions for the next trial without stopping the operation. This was important as the field trials were intended to replicate a real-life emergency scenario, wherein time is a critical component.

Equipment operators donned all necessary personal protective equipment including waterproof coveralls, rubber boots, hearing protection, eye protection, and for the field study trials, a safety harness. The water pump was switched on, pressurizing the fire hose. The operator of the foam nozzle carefully aimed the nozzle to the desired location to achieve a steady and even fill across the container. The nozzle lever was then rotated to the open position to produce foam. Operators ensured the fire hose did not become kinked as this would prevent adequate foam production. The lever was rotated to the closed position to discontinue foam production.

### Low- and high-pressure foam trials

The physical properties of each WBF were evaluated using both high- and low-pressure water pump systems. The quantified properties for each tested WBF were the ability to fill the container (yes/no), the time to fill the container (s), and the foam decay (cm/min) over 10 minutes, for three different foam concentrations (0.5, 1, and 3%).

**Fig 2:**
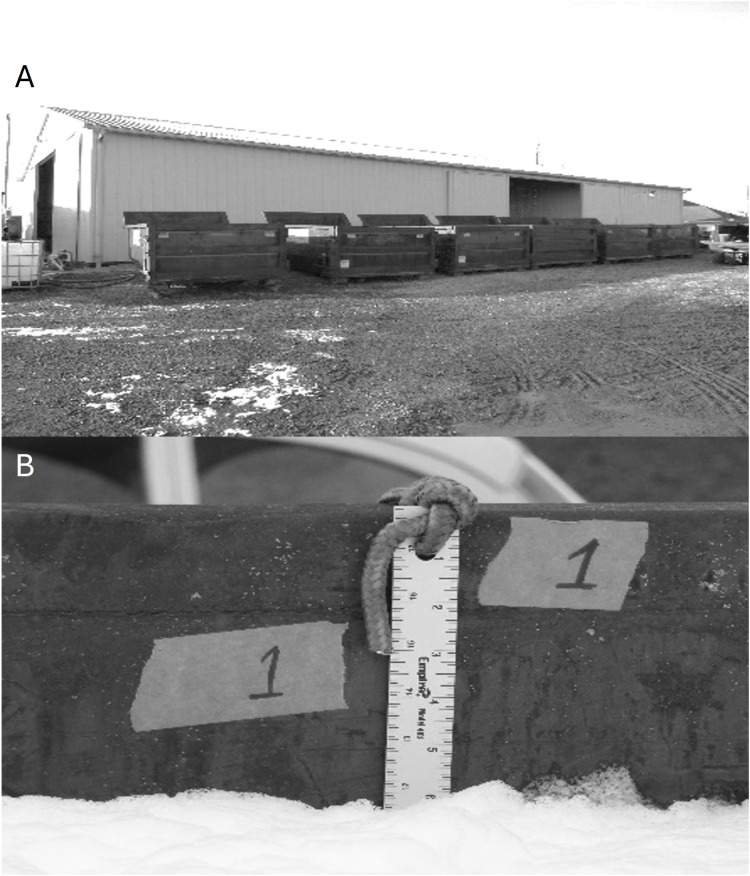
Equipment used for containing and measuring foam depth. Roll-off construction containers used to contain foam (A). Aluminum yard stick suspended into container for measuring foam height (B).

Six 11.5 m^3^ roll-off construction containers were used for these experiments (Fig 2A). A metal yard stick was vertically suspended from the top of the container by a rope. The yard stick was secured in place using magnets to prevent movement or floating during the filling process (Fig 2B). A 1% concentration of each foam concentrate was prepared as previously described pumped into the construction container. The pumps were stopped when the foam reached the top of the container. Filling was considered successful if the container was filled utilizing no more than 398 L of foam concentrate-water solution. For foams that did not completely fill the container, the filling deficit was determined by measuring the space between the foam surface and the top of the container. For all WBFs, filling success and filling time was recorded. After filling, the container was visually monitored for 10 minutes. The rate of decay of the foam product was determined by measuring the fall in the foam’s surface from the end of filling to 10-minutes post-filling, with measurements taken at 0, 5, and 10 minutes. At each time point, the suspended yard stick was used to measure the distance between the surface of the foam and the top of the container.

Foams that were successful at filling the container utilizing a 1% concentration were also tested at a 0.5% concentration. Foams that did not successfully fill the container at a 1% concentration were not retested at 0.5% as lower foam concentrate to water ratio results in reduced foam production. All WBFs were retested with a 3% concentration of the foam-water solution. Each WBF was tested in three replicates at each of the indicated concentrations. Dawn^®^ P&G Professional dish soap was evaluated using the same procedures with the high-pressure system. Because the concentrations had to be manually prepared and the tanks thoroughly rinsed between each use, the replicates for each concentration were conducted in sequence as triads. The 1% concentration was tested first, followed by 0.5%, (if the 1% fill was successful) and finally, the 3% concentration. The WBF concentrates with the best overall attributes, including successful and expedient fill times and minimal decay rates across all three concentrations, were selected for use in the exposure and field trials.

### Foam exposure trials

Exposure trials for each shortlisted Class A concentrate were conducted to evaluate potential indicators of behavioral or physiological distress to the pigs during depopulation.

Seventy-five mixed-sex, nursery-stage pigs (6–8-weeks old; average 13.1 kg) were locally sourced from a commercial nursery. The pigs were transported to the study site by the vendor and were temporarily housed and cared for according to specifications delineated in the Federation of Animal Science Societies Guide for the Care and Use of Agricultural Animals in Research and Teaching [37].

Each shortlisted Class A concentrate was tested in five replicates. Three pigs were used for each replicate (15 pigs per WBF, total n = 75). Three 1.46-m^3^ non-porous, commercial-grade polyethylene bulk containers (1.12 × 1.12 × 1.14 m, length, width, and height) were used to contain pigs during the depopulation process as previously used [11]. A single foaming equipment set up was assembled as previously described for the foam trials in this study. A custom-cut section of 0.64 cm thick plywood was fitted into the bottom of the container to provide a flat surface for the pigs to stand and to increase traction. A 1% concentration of WBF was pumped into the container until it was at the approximate shoulder height of the pigs (30–45 cm foam depth). Three pigs were selected by convenience and lowered by hand into the foam. The pigs were released once they gained traction on the bottom of the container and were able to stand unassisted. The pigs were then allowed to move around in the foam for 15 minutes. During this time, the pigs were observed for physiological parameters such as coughing, sneezing, or appearance of lesions to skin- or eyes. Behavioral parameters evaluated were vocalization, escape attempts, and rubbing skin or eyes. After 15 minutes, the depopulation protocol was initiated by applying additional foam to fill the container. The pigs were immersed and left in the foam undisturbed for a 7.5-minute dwell time, after which the container was emptied, and pigs were retrieved for physical and pathological examination. Death was confirmed by lack of respirations and absence of corneal reflex.

Immediately following depopulation of each replicate, partial necropsies were performed, focused on the cranial, cervical, and thoracic regions. Tissue samples from each pig were collected from the pinna, inguinal skin, palpebral conjunctiva, nasal turbinate, proximal trachea, and right cranial lung lobe. Tissues were grossly examined by trained veterinary pathologists for signs of erythema, edema, hyperemia, petechiae, ecchymoses, hemorrhage, or cyanosis (S1 Table). Additionally, the eyes were evaluated for scleral injection or corneal opacity and the lungs for congestion or emphysema. If gross changes were evident, samples representative of the most affected region were also collected for histopathology. The samples were immersed in 10% neutral buffered formalin for a fixation period of at least 72 hours and processed by The Ohio State University’s Comparative Pathology & Digital Imaging Shared Resource. Histologic slides were stained using hematoxylin and eosin. All slides were reviewed and scored by a resident in anatomic pathology according to the established rubric (S2 Table) under the guidance of two board-certified comparative anatomic pathologists.

### Foam field trials

The goal of the field trial was to evaluate the shortlisted Class A concentrates for efficacy and efficiency in producing rapid unconsciousness and subsequent death when applied to market-ready pigs in a real time, large-scale setting.

For the field trials, 411 mixed-sex, market-ready pigs (average weight = 127 kg, age = 16–17 weeks old) were obtained from a privately owned swine farm. These animals were all slated for depopulation due to accidental chemical contamination that rendered them unsuitable for human consumption. The animals had been cared for by the farm owner and staff following on-farm protocols commonly used in finishing-stage barns. For each of the shortlisted WBFs, a total of 50 pigs were tested in 3 replicates (n = 17, n = 17, n = 16, respectively) and three replicates of WD881 (n = 71, 65, and 68; total = 211) in a large-scale field trial scenario. A subset of five to seven randomly selected animals per replicate was implanted with subcutaneous dataloggers. The animals were briefly restrained, and local anesthesia was administered in the form of lidocaine HCL 2%, buffered with 8.4% sodium bicarbonate in a 1:10 ratio. Approximately 20–30 ml of the buffered solution (2–3 ml bicarbonate) was injected into the subcutaneous region of the body wall at the level of the elbow. A stab incision was made into the anesthetized tissue utilizing a #10 scalpel blade and extended 2–3 cm ventrally. The subcutaneous tissue was bluntly dissected either digitally or utilizing straight forceps, creating a pocket for the device. The data-logger (DST-Centi-HRT, Star-Oddi, Garðabær, Iceland) was inserted and seated into the pocket and the incision was closed using surgical skin staples. The devices were programed to record animal movement during the depopulation process.

A 64.5-m^3^ customized hydraulic trailer (12.2 × 2.36 × 2.24 m, length, width and height), previously described was used as the depopulation chamber [12]. The animals were moved directly from the barn onto the trailer via a loading ramp through the rear loading gate. Animals implanted with data-loggers were loaded first and allowed to randomly disperse among the other animals at will. The loading gate was secured, and the trailer was moved approximately 50 m to a relatively flat location to ensure that when foam was applied, the trailer was uniformly filled.

The trailer was then filled with 1% WBF. From the onset of the foam initiation, trained personnel monitored animal behavior until animals could no longer be observed in the rising foam. The number of vocalizations and escape attempts were documented. Additionally, the duration of animal movements was recorded, defined as the time between fill initiation and subsequent 7.5-minute dwell-time that visual or audible activity inside the trailer was observed. Once the dwell time elapsed, the trailer was moved to a designated disposal site and emptied. The animals were retrieved, and death was confirmed by lack of respirations and absence of corneal reflex. The animal movement duration was compared to animal cessation of movement as determined from data reported by the data-logger implants.

At the conclusion of the trials, the total cost of depopulation was estimated utilizing a custom-designed algorithm. By entering the number and size of pigs to be depopulated, size of the depopulation chamber, and cost of the foam product, the algorithm calculated the overall cost, broken down by cost of foam, water, and fuel required to depopulate the population.

### Time to cessation of movement

Due to foam coverage, pig activity was determined using external acceleration data from the implanted bio-loggers. The bio-logger measured the pig activity as tri-axial accelerations, or the intensity of movement in any spatial direction, which were analyzed according to methods previously outlined by Arruda et al. [11,12]. Baseline EA readings at rest were normalized to the standard acceleration of gravity (9.8 m/s^2^) and all deviations from this baseline were registered as a positive EA. A cessation of movement threshold was calculated for each pig individually. Clear voluntary pig activity was compared to the data point when any pig activity was no longer distinguishable from low EA data noise (i.e., wind effects on the trailer or false movements caused by activity by adjacent and still conscious pigs). Any EA value above the individually calculated threshold for each pig was considered indicative of pig movement. The COM was estimated using EA values between the initiation of the foam to the end of the 7.5-minute dwell time and calculated as the third quantile multiplied by 1.5 times the interquartile range, as established in prior studies [11,13,18,38]. The time to COM was defined as the difference between the first successive timepoint after the first EA value below the assigned threshold, with no successive values over threshold for a five-minute period, and the start of depopulation agent application [11].

### Statistical analysis

Statistical analyses were performed using GraphPad Prism®, Version 10.0.3 (275) (GraphPad Software Inc, Boston, Massachusetts) and Stata v17.0 (StataCorp, College Station, TX, USA). Statistical significance was declared at **P* *< 0.05 and any tendencies as 0.05 ≤ **P* *< 0.1.

#### Foam performance and exposure.

For the low- and high-pressure foam trials, the mean successful fill time and rate of decay across all replicates of each foam concentrate was compared to WD881 and between all groups using a one-way analysis of variance with Bonferroni corrections. Gross pathology and subsequent histologic findings were assigned a numerical score and compared utilizing Fisher’s exact test with pair-wise Bonferroni corrections.

#### Movement intensity.

The EA from 180 seconds (3 minutes) prior to the initiation of agent administration to 585 s (+10 min) post-agent administration was analyzed at 15 s intervals using a generalized linear mixed model with repeated measures for each method across each time point with pig nested within replicate as random effect. The EA data was transformed into normality using a log10 transformation. Model derived back transformed geometric means, and 95% confidence intervals were used for EA visualization. Post-hoc pairwise comparisons were performed to estimate the difference in EA between methods and Bonferroni correction was implemented to adjust for multiple tests. Total movement intensity throughout the depopulation process was calculated for each method across all replicates, using area under the curve between the first EA peak above to the last EA peak above a calculated geometric mean EA threshold (averaged across all methods). A one-way ANOVA was used to detect differences in mean total AUC between methods.

#### Time to cessation of movement.

Descriptive statistics, including mean, median, first and third quartile, interquartile range, standard deviation, were calculated and presented in text. A Kaplan-Meier survival curve with log-rank test was created to depict times to COM events per depopulation method. A linear regression model, with Bonferroni corrections to account for multiple comparisons, was used to investigate the effect of WBF products (BFN, BP, KD, PEF, and WD881) on times to COM. Depopulation method (WBF) was used as a fixed effect, with product as a random effect.

## Results

### High-pressure foam evaluation

Out of the foam concentrates tested, only four WBF’s (BFN, BP, KD, and PEF) besides WD881 successfully filled the container for all concentration levels tested (Table 2). Therefore, as this was the definition for shortlisting, only results for these four foams will be presented in comparison to WD881 below.

**Table 2:** Summary of mean fill time (seconds) and measure of success (Y = yes, N = no) for 100 gallons of high-pressure Class A foam concentrates at 0.5, 1, and 3% concentrations using a high-pressure pump system.

Class A Foam Concentrate	Mean Fill Time (s±SD)^a^			Container Fill Success (Y/N)		
	0.5%	1.0%	3.0%	0.5%	1%	3%
PHOS-CHEK WD881^b^	43.3 (3.2)	50.0 (3.5)	46.0 (1.0)	Y	Y	Y
Ansul Silv-ex Plus	−	−	45.0 (2.6)	-	N	Y
BioFor N^b^	78.7 (2.5)	49.3 (2.3)	43.0 (4.5)	Y	Y	Y
Buckeye Platinum^b^	51.7 (2.5)	51.3 (3.1)	50.3 (1.2)	Y	Y	Y
Chemguard Chemattack	−	64.7 (4.2)	56.7 (3.1)	N	Y	Y
Chemguard Class A Foam	−	45.7 (1.5)	52.0 (3.5)	N	Y	Y
Crestar	−	-	44.7 (2.5)	−	N	Y
Enforcer Firebull	−	−	46.0 (1.0)	−	N	Y
Fireade Class	−	−	47.3 (2.3)	−	N	Y
Firestopper AB 40002 FFC^c^	−	−	−	−	N	N
Fire-Trol 103^c^	−	−	56.0 (3.0)	N	N	Y
Knockdown^b^	48.0 (1.7)	52.7 (4.2)	52.3 (5.1)	Y	Y	Y
Pinnacle Class	−	−	−	−	N	N
Phos-Chek First response	−	−	52.0 (2.0)	−	N	Y
Polar Ecofoam^b^	71.0 (2.6)	50.3 (4.7)	56.7 (2.1)	Y	Y	Y
Solberg Fire-Brake	−	59.3 (6.1)	62.3 (0.6)	N	Y	Y
**Non-Class A Foam Concentrate**						
Dawn® dish soap	−	−	−	N	N	N

^a^If 1% concentration did not fill the container; the 0.5% concentration was omitted from testing.

^b^Short-listed foam concentrates for full assessment.

^c^Foams no longer in production at the time of publication

Missing data (–) indicates that data was not collected due to fill failure.

At 0.5% concentration, the mean (SD) fill times for BFN, BP, KD and PEF were slower compared to WD881. PEF and BFN filled the container significantly slower compared to WD881 (**P* *< 0.01) while KD tended to have a faster fill-time compared to WD881 (**P* *= 0.09). At 1% concentration, no significant differences in fill times were observed between the foams, but BFN had a numerically faster fill time compared to WD881 (Table 2).

For the 3% concentration the mean fill times for BP, KD and PEF were significantly slower compared to WD881 (*P* < 0.01) while no difference was observed between BFN and WD881 (Table 2). However, BFN was numerically faster compared to WD881 (Table 2).

The average decay rates for BFN, BP, KD, and PEF were faster compared to WD881 across 0.5%, 1% and 3% (Table 3). The decay rate of PEF was faster compared to WD881 at 0.5% (1.14 ± 0.3 vs. 0.36 ± 0.2 cm/minute, *P = *0.01) and 1% (1.5 ± 0.4 vs. 0.08 ± 0.2 cm/minute, **P* *< 0.01). The decay rate of BP was faster compared to WD881 at 3.0% concentration (0.46 ± 0.1 vs. 0.08 ± 0.2 cm/minute, **P* *= 0.02) (Table 3). No other significant differences in decay rate were observed.

**Table 3:** Average foam decay (inches/minute) for three foam-water solution concentrations (0.5%, 1% and 3%) for 16 Class A Foam produced in a high-pressure pump system. N/A represents unsuccessful foam fill attempts. Bold WBF indicates short-listed foams.

Company	Concentrate	Average foam decay (cm ± SD, per min)		
		0.5%	1%	3%
A.J Stone Company Ltd	Enforcer Firebull Class A Foam	n/a	n/a	0.69 ± 0.08
Ansul	Ansul Silv-ex Plus Class A Foam	n/a	n/a	0.18 ± 0.08
**BioEx**	**Bio For N Class A Foam**	**0.51 ± 0.25**	**0.25 ± 0.17**	**0.0 ± 0.0**
**Buckeye**	**Buckeye Platinum Class A Foam**	**0.46 ± 0.17**	**0.33 ± 0.35**	**0.18 ± 0.07**
Chemguard	Chemguard Chemattack Class A Foam	n/a	0.23 ± 0.28	0.64 ± 0.25
Chemguard	Chemguard Class A Plus Foam	n/a	1.70 ± 0.13	0.84 ± 0.33
Chemonics	Fire-Trol 103 Class A Foam	0.69	0.18 ± 0.28	0.36 ± 0.53
Crestar Fire	Crestar Class A Foam	n/a	n/a	0.33 ± 0.25
Dawn Dish Soap	Dawn detergent	n/a	n/a	n/a
Fireade	Fireade Class A Foam	n/a	n/a	0.38 ± 0.23
FireStopper, USA	Firestopper AB 40002 FFC Class A Foam	n/a	n/a	3.43 ± 2.92
**FireIce**	**Polar Ecofoam Class A Foam**	**1.14 ± 0.28**	**1.50 ± 0.43**	**0.56 ± 0.41**
Hazard Control Tech	Pinnacle Class A Foam	n/a	n/a	n/a
**National Foams**	**Knockdown Class A Foam**	**0.56 ± 0.08**	**0.08 ± 0.06**	**0.18 ± 0.06**
Perimeter Solutions	Phos-Chek First response Class A Foam	n/a	0.58 ± 0.53	0.15 ± 0.10
**Perimeter Solutions**	**Phos-Chek WD881 Class A Foam**	**0.36 ± 0.15**	**0.08 ± 0.15**	**0.08 ± 0.15**
Perimeter Solutions	Solberg Fire-Brake Class A Foam	n/a	0.74 ± 0.23	0.18 ± 0.03

Beyond the short-listed foam concentrates capable of working at all concentrations, an additional three products successfully filled the container at 1% concentration with a mean fill time of 57.1 s (± 8.6) and a decay rate of 1.1 (± 0.6) cm/minute. At 3% concentration, an additional nine foam concentrates successfully filled the container with a mean fill time of 52.5 s (± 6.1) and a decay rate of 0.5 (± 0.3) cm/minute. Dawn^®^ dish soap was unable to generate enough foam to fill the container at any concentration level and was not further considered. Mean fill time and fill success for all individual foams and concentrations for the high-pressure trial are listed in Table 2.

### Low-pressure foam evaluation

Fewer foam concentrates were used for the low-pressure foam evaluation as some foams were no longer in production at the time of testing. Due to the results obtained for Dawn^®^ dish soap in the high-pressure trial, this product was left out for further testing. Out of the foam concentrates tested, only two WBF’s (BP and KD) besides WD881 successfully filled the container for all concentration levels tested (0.5%, 1%, and 3%) (Table 4). Therefore, as this was the definition for shortlisting, only results for these three foams will be presented in comparison to WD881 below. At 0.5% concentration, the mean (SD) fill times for BP and KD were 93.3s (±1.5) and 113.0s (14.7) respectively, compared to 149.3s (±27.8) for WD881 (Table 4). At 1% concentration, the mean fill time for BP and KD, were 99.7s (±3.8) and 106.7s (6.5) respectively, compared to 99.0s (± 1.7) for WD881 (Table 4). For the 0.5% concentration the fill time for BP was significantly faster compared to WD881 (*P* = 0.03) and tended to be faster compared to KD (**P* *= 0.08). No difference in fill time between KD and WD881 was observed. For the 3% concentration the mean fill time for BP and KD was 100.3s (±0.6) and 111.0s (8.9) respectively, compared to 108.0s (± 1.7) for WD881 (Table 3). Regarding individual WBF fill times, BP filled the container significantly faster compared to WD881 (**P* *< 0.01) while no difference was observed between KD and WD881 (**P* *= 0.11) at the 3% concentration (Table 4). No significant differences in fill times were observed at 1% concentration.

**Table 4:** Summary of mean fill time (seconds) and measure of success (Y = yes, N = no) for 100 gallon of high-pressure Class A foam concentrates at 0.5, 1, and 3% concentrations using a low-pressure pump system.

Class A Foam Concentrate	Mean Fill Time (s ± SD)^a^			Container Fill Success (Y/N)		
	0.5%	1%	3%	0.5%	1%	3%
PHOS-CHEK WD881	152.7 (22.5)	99.0 (6.1)	113.7 (9.8)	Y	Y	Y
Ansul Silv-ex Plus	–	–	112.3 (11.8)	–	N	Y
BioFor N	–	109.7 (13.8)	108.0 (12.2)	N	Y	Y
Buckeye Platinum	46.3 (0.6)	103.0 (4.6)	100.3 (0.6)	Y	Y	Y
Chemguard Chemattack	–	125.7 (15.5)	108.7 (9.0)	N	Y	Y
Chemguard Class A Foam	–	102.0 (8.3)	111.3 (9.7)	N	Y	Y
Crestar	–	–	108.7 (3.0)	–	N	Y
Enforcer Firebull	–	–	–	–	N	N
Knockdown	113.0 (14.7)	106.7 (6.5)	111.0 (8.9)	Y	Y	Y
Pinnacle Class	–	–	–	–	N	N
Phos-Chek First response	–	144.0 (12.6)	112.3 (10.9)	N	Y	Y
Polar Ecofoam	–	128.0 (7.8)	120.7 (14.3)	N	Y	Y
Solberg Fire-Brake	–	–	110.0 (8.0)	N	N	Y

^a^If the 1% concentration did not fill the container; the 0.5% concentration was omitted from testing.

^b^Foams no longer in production at the time of publication

Missing data (–) indicates that data was not collected due to fill failure.

The decay rate of WD881 was faster compared to BP at 0.5% (1.78 ± 0.3 vs. 0.58 ± 0.2 cm/minute, *P = *0.01) and 1% (0.97 ± 0.4 vs. 0.43 ± 0.2 cm/minute, **P* *< 0.01) (Table 5). No other statistical differences between the short-listed WBFs were observed.

**Table 5:** Average foam decay (inches/minute) for three foam-water solution concentrations (0.5%, 1% and 3%) for 16 Class A Foam produced in a low-pressure pump system. N/A represents unsuccessful foam-fill attempts. Bold WBF indicates short-listed foams.

Company	Concentrate	Total foam decay (cm per minute)		
		0.5%	1%	3%
A.J Stone Company Ltd	Enforcer Firebull Class A Foam	n/a	n/a	n/a
Ansul	Ansul Silv-ex Plus Class A Foam	n/a	n/a	0.51 ± 0.25
BioEx	Bio For N Class A Foam	n/a	0.0 ± 0.0	0.08 ± 0.15
**Buckeye**	**Buckeye Platinum Class A Foam**	**0.58 ± 0.15**	**0.43 ± 0.15**	**0.33 **±** 0.15**
Chemguard	Chemguard Chemattack Class A Foam	n/a	1.19 ± 0.89	1.19 ± 0.53
Chemguard	Chemguard Class A Plus Foam	n/a	3.30 ± 0.66	2.54 ± 0.25
Crestar Fire	Crestar Class A Foam	n/a	n/a	0.84 ± 0.56
FireIce	Polar Ecofoam Class A Foam	n/a	1.57 ± 0.46	2.08 ± 0.08
Hazard Control Tech	Pinnacle Class A Foam	n/a	n/a	n/a
**National Foams**	**Knockdown Class A Foam**	**1.09 ± 0.56**	**0.33 ± 0.15**	**0.84 ± 0.28**
Perimeter Solutions	Phos-Chek First Response Class A Foam	n/a	1.27 ± 0.25	0.25 ± 0.43
**Perimeter Solutions**	**Phos-Chek WD881 Class A Foam**	**1.78 ± 1.55**	**0.97 ± 0.48**	**0.43 ± 0.28**
Perimeter Solutions	Solberg Fire-Brake Class A Foam	n/a	n/a	0.25 ± 0.43

Beyond the short-listed WBFs capable of performing well at all concentrations, an additional four foam concentrates (Chemguard Chemattack, BFN, Phos-Chek First Response and PEF) successfully filled the container at 1% and 3% concentrations (Table 3). The mean fill times for these foams were 126.8 s (± 16.2) and 112.4 s (± 9.0) with a decay rate of 1.0 (± 0.8) and 0.9 (± 0.4) cm/minute for 1% and 3% concentrations, respectively. Mean fill times and fill successes for all individual foams and concentrations for the high-pressure trial are listed in Table 3. As BP and KD also were successful in the high-pressure pump system trial, only high-pressure pump systems foams were carried over to field-trials due to the faster fill times.

### Exposure trials

The nursery-aged pigs used in this portion of the experiment vocalized while being physically handled, however, they rapidly calmed once lowered into the foam (approximately shoulder depth) and released. During the 15-minute exposure period, the pigs stood still or moved about calmly. No physiological changes (coughing, sneezing, redness in skin or eyes) or behavioral signs of distress or aversion to the foam (vocalizing, escape attempts, rubbing skin or eyes) were observed for any of the WBF concentrates. Following the 7.5-minute dwell period, all animals were dead with no signs of corneal reflex, or respiratory activity upon examination.

The major gross findings were congestion in the right cranial lung lobe and the presence of cyanosis. Collectively, 45.3% (34/75) of the animals had some degree of lung congestion and 41.3% (31/75) had some degree of cyanosis. Only 20% (15/75) showed signs of both cyanosis and lung congestion and 30.6% (23/75) animals did not have either lesion. By Fischer’s exact test, there was no significant association between the two lesions in any of the tested products; likewise, overall, no significant difference was observed between the groups (*P = *0.82). Mild erythema was observed in the nasal mucosa of only one pig in the WD881 group. Two pigs in the Knockdown group had gross lesions deemed to be unrelated to the experiment including an umbilical abscess and mild subcutaneous edema in the inguinal region. No other gross lesions were noted among any of the foam concentrates.

The major microscopic finding was pulmonary hemorrhage, which was observed in at least one pig for each of the WBFs. The distribution of these hemorrhages was predominantly perivascular within the minimally to mildly affected pigs, with extension into peribronchiolar regions and alveolar spaces with increasing severity (S1 Fig). The degree of pulmonary hemorrhages was significantly different between foam groups, based on Fischer’s exact test across all treatment groups (*P = *0.04). The pair-wise comparison revealed that pigs depopulated with BioFor N had fewer hemorrhages with a score of three or four compared to WD881 (*P = *0.04).

Inflammatory infiltrates were occasionally observed but no significant differences across treatment groups within nasal turbinates (*P = *0.75), the trachea (*P = *0.52), or the conjunctiva (*P = *0.39) were detected. Dermal changes were overall minimal to mild, and there were no significant differences across treatment groups for both pinna (*P = *0.34) and inguinal skin (*P = *0.96).

### Field trials

**Fig 3:**
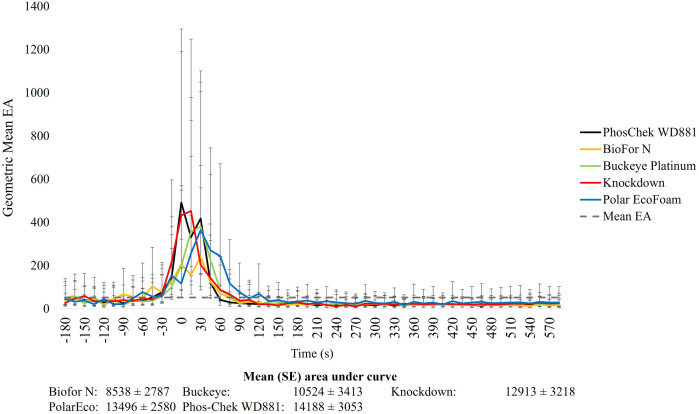
Mean [95% confidence interval] pig activity (EA) for pigs depopulated with medium-expansion water-based Class-A foams (BioFor N, Buckeye Platinum, Knockdown, Polar EcoFoam, and Phos-Chek WD881). Foam initiation occurred at time point zero. Mean (±SE) area under the curve represents the average total activity performed for all animals across all replicated for each foam. Mean geometric EA across all foams and replicates is indicated by the dotted black line.

**Fig 4:**
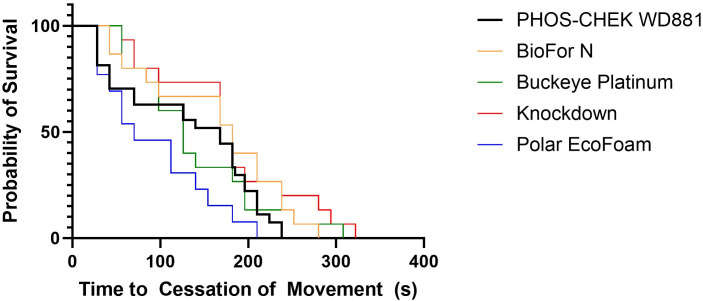
Probability (%) of swine survival, determined by the time (s) to cessation of movement, when exposed to water-based Class A foam concentrates during depopulation.

The fill time varied among the shortlisted test foams, with an average (±SD) fill of 81.3 s (±20.5). WD881 filled the fastest in 61.1 s (±5.5), while the other WBFs ranged from 58.3 s (±3.1) (KD) to 105.3s (± 9.8) (PEF). The average time to last observed activity above the calculated threshold for the test foams was 121.5 s (±11.0) compared to 137.8 s (±25.7) observed with WD881. No significant EA differences were observed between the shortlisted foams and WD881 throughout the depopulation procedure (Fig 3). Similarly for all products, pigs reacted to the initiation of foam which continued throughout the filling process up until approximately 120–150 s post fill-initiation (Fig 3). Additionally, no significant differences in AUC were observed between shortlisted foams (Fig 3). All the tested WBFs produced rapid COM (Fig 4). No statistical difference was observed for the mean time (s ± SE) to COM between the WBFs (210.7 ± 34.2, 112.9 ± 24.3, 116.2 ± 17.3, 128.8 ± 15.6, and 186.9 ± 40.0 s, for WD881, BFN, PEF, BP and KD, respectively, *P > *0.05). However, data from the third replicate for PEF is not reported and is omitted from statistical evaluation because the biologger did not capture the entire dwell time. The manually recorded time to last animal movement time was significantly longer for KD and BFN, compared to COM estimates (*P = *0.01 and *P < *0.01 respectively). There was no difference between the manually recorded time to the last animal movement and COM estimates for WD881 (*P = *0.24), BP (*P = *0.23), or PE (*P = *0.47). The shortlisted WBFs successfully completed the depopulation process and 100% of swine were deceased with no possibility of resuscitation after the 7.5-minute dwell time.

A custom-designed algorithm for determining the cost of depopulation was used to estimate the cost for depopulation. The calculation computes the total cost through analysis of the number of swine to be depopulated, the size of the depopulation chamber, the cost of fuel, and the cost of the WBF used. Calculation of the cost to depopulate 1000-head of market ready swine ranged from $936.20 (KD) to $1,608.80 (BP) with a mean of $1,237 ± 311.5 (Table 6). This estimate did not account for start-up costs that include the purchase of foaming equipment for disposal of animals following depopulation.

**Table 6:** Summary of estimated cost of depopulation ($USD) of 1000-head of market-ready swine using different Class A water-based foam concentrates. Estimated cost includes foam, water, and fuel. It does not include additional costs for shipping, labor, or disposal.

Class A Concentrate	Estimated Depopulation Cost ($ USD)
PHOS-CHEK WD881	1501.1
Buckeye Platinum	1608.8
Polar EcoFoam	1197.9
BioFor N	940.2
Knockdown	936.2

## Discussion

The results of this study illustrate that the physical properties and subsequent suitability as WBF depopulation agents differ greatly based on brand or manufacturer of the product. It was determined that not all products were suitable for depopulation of swine utilizing the equipment repertoire utilized in this study. Many of the tested products did not produce adequate foam or may require higher WBF concentrations or modifications to the delivery system to be effective. Until more is known about the performance capabilities of these WBFs, these products should be used with caution and if used, should be assessed by the end user for suitability prior to deployment for depopulation. However, in addition to PHOS-CHEK® WD881, four Class A water-based foam products, BioFor N®, Buckeye® Platinum, Knockdown®, and Polar® EcoFoam, were identified as suitable for emergency swine depopulation using this study’s foaming equipment.

### Foam trials

The chemical composition of a WBF product determines the foam stability and the rate at which foam decays. Surfactant molecules increase the surface tension of the bubble and improve stability of medium- to high-expansion foams as moderately sized bubbles are more dry and slow-draining [39–41]. Studies have shown that the degree of expansion, bubble size, and permeability are impacted by the composition and molecular weight of surfactants in the WBF [39,42]. In this study, the chemical composition of the tested products was not analyzed. However, it is suspected that these properties contributed to the failure of some WBFs’ ability to fill a container. Thus, it’s possible that chemical analyses alone could be used as a screening tool to identify key foam properties in existing products prior to full scale testing. Across the successful products in this study, the decay rates were negligible. The greatest difference between these was less than 0.6 cm/min, at which the foam level would not go below a depth of at least twice the height of the animals for the duration of the dwell time. Previous studies have shown that increased concentrations improve foam stability. It is also known that differences in application modality and mix ratio directly impact the efficacy of WBF products on fire suppression [17,41]. Although many of the tested WBFs did not produce stable foam for the purpose of this study, it is possible that these products could be effective for depopulation if used in higher concentrations (≥3%) or with different equipment. Future studies evaluating different foam generation equipment and associated parameters will likely provide more options for emergency depopulation of swine using WBF.

### Exposure trials

The shortlisted WBF products that were tested (BFN, BP, KD, and PEF), in addition to WD881, contain anionic surfactants, alcohols, and detergents, all of which are known to cause skin and eye irritation in addition to being harmful if swallowed [20–23,36,43]. Dermatologic lesions and discomfort have been associated with disruption of the epithelial barrier and surfactant absorption from exposure to detergents, surfactants, and alcohols [44–46]. Consequently, less irritating compounds, including sugar-based and those with a more neutral pH, are being investigated [47–49]. In the current study, the pigs appeared to be unaffected by the foam, likely because they were exposed to very low concentrations of WBF during a short time frame. These findings are consistent with another study wherein only high surfactant concentrations caused irreversible corneal damage (>10%) and acute toxicity (>30%) in rabbits [50]. The chemical components of the shortlisted WBFs, though proprietary, contain surfactants that are thought to be mild, resulting in subjectively normal behavior and minimal irritation and distress during the exposure period. Combining these with the observations of Korenyi-Both et al. (2022), who demonstrated unconsciousness in nursery pigs within approximately two minutes of application of WBF, it was concluded that WBF has minimal effect on animal behavior and irritation of skin, eyes, mucous membranes and lungs during the brief period between exposure and unconsciousness [13].

Following depopulation, some of the pigs appeared cyanotic which is consistent with hypoxic death. Foam causes obstruction of the upper airways, leading to asphyxia, hypoxia, and rapid loss of consciousness. Obstructive hypoxia has been reported in children along with cyanosis, pulmonary hemorrhage, and emphysema, which are typical findings in these cases [51]. Park, et. al. reported similar findings indicative of occusive hypoxia in swine depopulated utilizing both WBF and nitrogen-based foam [52]. Upon microscopic evaluation, pulmonary hemorrhages were consistent with the petechial to ecchymotic hemorrhages noted on gross examinations. Pulmonary hemorrhage is the only microscopic lesion with significant differences among the shortlisted WBF concentrates. However, given the presence of this change in all the trials, the pulmonary hemorrhages may be related to increased perimortem dyspnea, rather than a result of a particular WBF product. Inflammation within the nasal turbinates, trachea, conjunctiva, and skin was noted on gross examination. Inflammation is a non-specific finding that can be associated with exposure to WBF, infection, allergy, or environmental exposure to noxious substances, such as ammonia. Exposure to ammonia can lead to inflammation of the respiratory epithelium which has been reported in pigs exposed to 25 ppm of NH_3_ [53,54]. Because significant differences in inflammation were not observed, these findings are suspected to be attributed to underlying etiologies, including chronic exposure to low levels of harmful NH_3_ gas, rather than short-term exposure to water-based foam.

### Field trials

In this study, a large-scale, real-time depopulation event demonstrated that WD881 and all the short-listed foam concentrates were effective for depopulation of market-ready swine. This is not surprising for WD881, which has been equally effective for depopulation of piglets, finisher pigs, sows, cattle, and poultry [2,3,11,12,15,19,55,56]. No significant differences in EA, AUC, or COM between the short-listed concentrates and WD881 were observed, indicating that, overall, the products performed comparably in time for inducing unconsciousness and death. PEF appears to have the shortest time to COM which may in part be due to some missing data, but until chemical analyses are conducted, the effect of specific chemical compositions or foam qualities can’t be fully disregarded. The work of Korenyi-Both et al. demonstrated loss of consciousness in swine to be no more than 2 min, 19 seconds using electroencephalogram during WBF depopulation, which supports the EA and COM data reported in the present study [13].

In addition to establishing the efficiency of selected products, the cost of the short-listed WBFs were similar to WD881 made them suitable candidates also from a financial standpoint. Costs associated with shipping, equipment acquisition, labor, and disposal were not evaluated due to expected variances among geographical regions. The cost of the WBF product itself was the primary influencing factor in the cost estimates. However, based on the data from the foam trial studies, it may be possible to use any of the shortlisted WBFs at lower concentrations (0.5%), especially if funding or supplies are limited. This would reduce the required volume of foam concentrate and decrease the overall cost of an emergency depopulation event; however, it may have negative animal welfare implications due to longer fill times. Although a custom-built depopulation trailer was employed for this study, cost-saving options include rental dumpsters, or making modifications to existing trailers [12,57]. Future cost analyses focused on estimation of variable costs in high pork-producing regions would provide a more accurate picture of economic impact to identify additional methods for minimizing the financial loss associated with depopulation of swine.

### Study limitations

The selection of WBF products was limited to those which were available for purchase and shipping with the geographic region. Due to differences in market availability, other options could be available. The replicates in each trial were conducted consecutively and animals were assigned to each of the WBF groups based on practicality rather than random selection. It is important to note that cessation of movement data was used as an analog for unconsciousness and not an indicator of death. It is also understood that the WBF products tested in this study may perform differently if applied using alternate equipment configurations or in varying environmental contexts. Finally, gross and histopathological interpretation may have been influenced by the variable time to necropsy and sample collection, post-mortem movement, or multiple individuals providing observations.

## Conclusions

The current study assessed 15 commercially available WBF products based physical attributes and decay rate. It was determined that multiple products could be used for successful depopulation of swine by employing both high- and low-pressure foaming systems. Furthermore, products were identified that, depending on conditions, may not be suitable for depopulation purposes. This study also evaluated the physiological effects and degree of swine aversion following short-term exposure to the shortlisted WBFs during the depopulation process and determined them to be minimal. Finally, the current study demonstrated the efficacy of the shortlisted WBFs in a large-scale depopulation scenario and successfully estimated the overall cost of depopulation.

This study effectively expanded the options available for WBF depopulation of swine. This will improve the national availability of this method and reduce supply chain bottlenecks and intra-industry competition for resources during simultaneous multi-site depopulation events. Investigations focused on availability and efficacy of various foam-generation equipment in addition to regionally applicable cost analyses are subsequent actions to be taken to further refine WBF depopulation options and improve overall emergency response capabilities during emergency situations such as a foreign animal disease outbreak.

## Supporting information

S1 Fig Representative photomicrographs of pulmonary hemorrhages in nursery pigs depopulated using Class A foam concentrate by score. **A)** Score 0, where no hemorrhage is present within pulmonary parenchyma. H&E, 40X. **B)** Score 1, where hemorrhages affect <10% of parenchyma. H&E, 40X. **C)** Score 2, where hemorrhages affect >10–25% of parenchyma. H&E, 40X. **D)** Score 3, where hemorrhages affect >25–50% of parenchyma. H&E, 40X. **E)** Score 4, where hemorrhages are present >50% of parenchyma. H&E, 40X. **F)** The distribution of hemorrhages within the most severely affected lungs consisted of a perivascular, peribronchiolar, and interalveolar pattern. H&E, 100X.
(TIF)

S1 Table Rubric for gross pathological assessment of sampled tissues from pigs from exposure trial experiments. (PDF)

S2 Table Rubric for histological assessment of sampled organs from pigs from exposure trial experiments. (PDF)

## Abbreviations

ANOVA: analysis of variance
AUC: area under the curve
BFN: Bio For N
BP: Buckeye Platinum
COM: cessation of movement
CO2: carbon dioxide gas
EA: external acceleration
IBC: intermediate bulk container
KD: Knockdown
NH3: ammonia
NVS: National Veterinary Stockpile
PEF: Polar EcoFoam
SD: standard deviation
WBF: Class A water-based foam
WD881: PHOS-CHEK WD881

## References

[1] Leary S, et al. AVMA guidelines for the depopulation of animals: 2019 edition. 2019 ed. Schaumburg, IL: American Veterinary Medical Association. 2019.

[2] Benson ER, Alphin RL, Rankin MK, Caputo MP, Hougentogler DP, Johnson AL. Mass emergency water-based foam depopulation of poultry. Avian Dis. 2012;56(4 Suppl):891–6. doi: 10.1637/10160-040912-Reg.1 23402109

[3] Rankin MK, Alphin RL, Benson ER, Johnson AL, Hougentogler DP, Mohankumar P. Comparison of water-based foam and carbon dioxide gas emergency depopulation methods of turkeys. Poult Sci. 2013;92(12):3144–8. doi: 10.3382/ps.2013-03341 24235223

[4] Kaiser M, Kristensen JK, Thomsen PT. Technical note: Construction of a CO 2 supply system for depopulation of pigs in a container. Transl Anim Sci. 2025;9:txaf034. doi: 10.1093/tas/txaf034 40264540 PMC12012665

[5] Anderson KN, Deen J, Karczewski J, Zhitnitskiy PE, Vogel KD. History and best practices of captive bolt euthanasia for swine. Transl Anim Sci. 2022;6(2):txac065. doi: 10.1093/tas/txac065 35755133 PMC9217757

[6] Casey-Trott TM, Millman ST, Turner PV, Nykamp SG, Lawlis PC, Widowski TM. Effectiveness of a nonpenetrating captive bolt for euthanasia of 3 kg to 9 kg pigs. J Anim Sci. 2014;92(11):5166–74. doi: 10.2527/jas.2014-7980 25349360

[7] Baysinger A, Kogan LR. Mental Health Impact of Mass Depopulation of Swine on Veterinarians During COVID-19 Infrastructure Breakdown. Front Vet Sci. 2022;9:842585. doi: 10.3389/fvets.2022.842585 35450138 PMC9016222

[8] Kollias NS, et al. A literature review on current practices, knowledge, and viewpoints on pentobarbital euthanasia performed by veterinarians and animal remains disposal in the United States. Journal of the American Veterinary Medical Association. 2023;6. 10.2460/javma.22.08.0373 36800298

[9] Arluke A. Managing emotions in an animal shelter. In: Manning A, Serpell J. Animals & Society: Changing Perspectives. London: Routledge. 1994. 145–56.

[10] Johnson AK, Rademacher CJ, Eggers J, Gabler NK, Greiner LL, Kaisand J, et al. Innovative strategies for managing swine welfare during the COVID-19 pandemic in Iowa. Transl Anim Sci. 2021;5(4):txab225. doi: 10.1093/tas/txab225 34993422 PMC8722373

[11] Kieffer JD, Campler MR, Cheng T-Y, Arruda AG, Youngblood B, Moeller SJ, et al. Evaluation of a Water-Based Medium-Expansion Foam Depopulation Method in Suckling and Finisher Pigs. Animals (Basel). 2022;12(8):1041. doi: 10.3390/ani12081041 35454287 PMC9027019

[12] Arruda AG, Campler MR, Cheng T-Y, Youngblood B, Capria V, Kieffer J, et al. Reliability of water-based medium-expansion foam as a depopulation method for nursery pigs and cull sows. Transbound Emerg Dis. 2022;69(5):e2719–30. doi: 10.1111/tbed.14622 35691016 PMC9796781

[13] Korenyi-Both J, Vidaurre J, Held T, Campler MR, Kieffer J, Cheng T-Y, et al. Description of electroencephalographic data gathered using water-based medium-expansion foam as a depopulation method for nursery pigs. Sci Rep. 2022;12(1):16798. doi: 10.1038/s41598-022-21353-7 36207428 PMC9546833

[14] Lorbach JN, Campler MR, Youngblood B, Farnell MB, Beyene TJ, Kieffer J, et al. Comparison of Gaseous and Water-Based Medium-Expansion Foam Depopulation Methods in Cull Sows. Animals (Basel). 2021;11(11):3179. doi: 10.3390/ani11113179 34827911 PMC8614275

[15] Capria VM, Arruda AG, Cheng T-Y, Campler MR, Youngblood BL, Moeller SJ, et al. Water-based medium-expansion foam depopulation of adult cattle. Transl Anim Sci. 2023;7(1):txad065. doi: 10.1093/tas/txad065 37415594 PMC10321402

[16] Fan YY, et al. Rheological characterization and prediction model of compressed air Class A foam. Journal of Dispersion Science and Technology. 2022;43(7):13.

[17] Management F.a.A. Class A foam for wildland fire management. Washington, DC: US Forest Service. 2018.

[18] Campler MR, Cheng T-Y, Arruda AG, Flint M, Kieffer JD, Youngblood B, et al. Refinement of water-based foam depopulation procedures for finisher pigs during field conditions: Welfare implications and logistical aspects. Prev Vet Med. 2023;217:105974. doi: 10.1016/j.prevetmed.2023.105974 37423152

[19] Benson ER, Alphin RL, Dawson MD, Malone GW. Use of water-based foam to depopulate ducks and other species. Poult Sci. 2009;88(5):904–10. doi: 10.3382/ps.2008-00268 19359675

[20] Safety Data Sheet PHOS-CHEK-WD881. Green Bay, WI: Perimeter Solutions; 2023.

[21] Bio for N safety data sheet. Sainte-Consorce, France: BIOEx. 2020.

[22] FireIce Polar EcoFoam Class A Safety Data Sheet. GelTech Solutions. 2018.

[23] *KnockDown ® Class “A” Foam Concentrate Safety Data Sheet*. 2019, National Foam: West Chester, PA; 8.

[24] Dawn dish detergent safety data sheet. Cincinnati, OH: Procter and Gamble, Inc. 2015.

[25] Phos-Chek First Response Safety Data Sheet. Green Bay, WI: Perimeter Solutions. 2022.

[26] Solberg fire-brake safety data sheet. St. Marys, Australia: Solberg Asia Pacific Pty Ltd. 2015.

[27] FireTrol safety data sheet. Phoenix, AZ: Fire-Trol Holdings, L.L.C. . 2000.

[28] FireStopper International Limited. Firestopper AB 40002 (FFC) Safety Data Sheet. 2016.

[29] Fireade mil 3% AFFF fire fighting foam safety data sheet. Fayette, GA: Fire Service Plus, Inc. 2017.

[30] FIREBULL F3 Fluorine Free Foam Safety Data Sheet. Peachtree City, GA: EnforcerOne, LLC. 2020.

[31] CHEMGUARD Class A Plus CA Material Safety Data Sheet. 2013, Chemguard, Inc.: Mansfield, TX; 6.

[32] SILV-EX PLUS Class A Fire Control Foam Concentrate Safety Data Sheet. Marinette, WI: Tyco Fire Protection Products. 2015.

[33] Crestar material safety data sheet. Smithville, OH: Crestar Firefighting Equipment LLC. 2010.

[34] Chemguard DirectAttack Class A Foam Safety Data Sheet. Marinette, WI: Tyco Fire Protection Products. 2016.

[35] Pinnacle Class A Foam Safety Data Sheet. Fayetteville, GA: Hazard Control Technologies, Inc. 2023.

[36] Equipment B.F. Safety data sheet Buckeye Platinum 1% AFFF. Kings Mountain, NC: Buckeye Fire Equipment Company. 2015.

[37] Tucker CB, MacNeil MD, Webster AB. Guide for the care and use of agricultural animals in research and teaching. 8th ed ed. American Dairy Science Association, American Society of Animal Science, Poultry Science Association. 2020.

[38] Arruda AG, Beyene TJ, Kieffer J, Lorbach JN, Moeller S, Bowman AS. A Systematic Literature Review on Depopulation Methods for Swine. Animals (Basel). 2020;10(11):2161. doi: 10.3390/ani10112161 33233523 PMC7699593

[39] Davis M. Basic physics of foam stability and collapse. Naval Fuels & Lubricants. Patuxent River, MD. 2012. 24.

[40] Laundess AJ, Rayson MS, Dlugogorski BZ, Kennedy EM. Small-Scale Test Protocol for Firefighting Foams DEF(AUST)5706: Effect of Bubble Size Distribution and Expansion Ratio. Fire Technol. 2011;47(1):149–62. doi: 10.1007/s10694-009-0136-2

[41] Xu Z, Guo X, Yan L, Kang W. Fire-extinguishing performance and mechanism of aqueous film-forming foam in diesel pool fire. Case Studies in Thermal Engineering. 2020;17:100578. doi: 10.1016/j.csite.2019.100578

[42] Laundess AJ, et al. Suppression performance comparison for aspirated, compressed-air and in situ chemically generated Class B foams. Fire Technology. 2012;48(3):17.

[43] Bio for N wetting and foaming additive technical data sheet. Sainte Consorce, France: BIOEx. 2021.

[44] Chopin-Doroteo M, Krötzsch E. Soap or alcohol-based products? The effect of hand hygiene on skin characteristics during the COVID-19 pandemic. J Cosmet Dermatol. 2023;22(2):347–53. doi: 10.1111/jocd.15523 36409429

[45] Bois R, et al. Physicochemical, foaming and biological properties of lowly irritant anionic sugar-based surfactants. Colloids and Surfaces A: Physicochemical and Engineering Aspects. 2020;607:9.

[46] Slotosch CM, Kampf G, Löffler H. Effects of disinfectants and detergents on skin irritation. Contact Dermatitis. 2007;57(4):235–41. doi: 10.1111/j.1600-0536.2007.01200.x 17868216

[47] Sonoda J, et al. Skin penetration of fatty acids from soap surfactants in cleansers dependent on foam bubble size. Journal of Surfactants and Detergents. 2014;17(1):7.

[48] Hawkins S, Dasgupta BR, Ananthapadmanabhan KP. Role of pH in skin cleansing. Int J Cosmet Sci. 2021;43(4):474–83. doi: 10.1111/ics.12721 34137035

[49] Fei D, Zhou G, Yu Z, Gang H, Liu J, Yang S, et al. Low‐Toxic and Nonirritant Biosurfactant Surfactin and its Performances in Detergent Formulations. J Surfact & Detergents. 2020;23(1):109–18. doi: 10.1002/jsde.12356

[50] Wibbertmann A, Mangelsdorf I, Gamon K, Sedlak R. Toxicological properties and risk assessment of the anionic surfactants category: Alkyl sulfates, primary alkane sulfonates, and α-olefin sulfonates. Ecotoxicol Environ Saf. 2011;74(5):1089–106. doi: 10.1016/j.ecoenv.2011.02.007 21463896

[51] Lavrukova OS, Polyakov АYu, Beraya RF, Popov VL. To the problem of death of children from closure of respiratory tract by foreign object: expert observation. Russian Journal of Forensic Medicine. 2022;8(1):25–30. doi: 10.17816/fm682

[52] Park JY, Campler MR, Cheng T-Y, Youngblood BL, Torrisi D, Cressman MD, et al. Assessment of three large-scale depopulation methods for swine. PLoS One. 2025;20(3):e0320217. doi: 10.1371/journal.pone.0320217 40132003 PMC11936211

[53] Wang H, Zeng X, Zhang X, Liu H, Xing H. Ammonia exposure induces oxidative stress and inflammation by destroying the microtubule structures and the balance of solute carriers in the trachea of pigs. Ecotoxicol Environ Saf. 2021;212:111974. doi: 10.1016/j.ecoenv.2021.111974 33508713

[54] Urbain B, Gustin P, Charlier G, Coignoul F, Lambotte JL, Grignon G, et al. A morphometric and functional study of the toxicity of atmospheric ammonia in the extrathoracic airways in pigs. Vet Res Commun. 1996;20(4):381–99. doi: 10.1007/BF00366545 8865581

[55] Caputo MP, Benson ER, Pritchett EM, Hougentogler DP, Jain P, Patil C, et al. Comparison of water-based foam and carbon dioxide gas mass emergency depopulation of White Pekin ducks. Poult Sci. 2012;91(12):3057–64. doi: 10.3382/ps.2012-02514 23155013

[56] Benson E, Malone GW, Alphin RL, Dawson MD, Pope CR, Van Wicklen GL. Foam-based mass emergency depopulation of floor-reared meat-type poultry operations. Poult Sci. 2007;86(2):219–24. doi: 10.1093/ps/86.2.219 17234833

[57] Pepin B, Williams T, Odland C, Spronk T, Nerem J. Modification of a standard dump trailer into a mobile carbon dioxide depopulation unit for swine. JSHAP. 2022;30(1):31–8. doi: 10.54846/jshap/1243

