# Supplementary figures and images for: Evaluation of commercially available class A water-based foam concentrates for swine depopulation
Source: PLoS One. 2025 Aug 18;20(8):e0328073. doi: 10.1371/journal.pone.0328073 (PMC12360584; doi:10.1371/journal.pone.0328073)

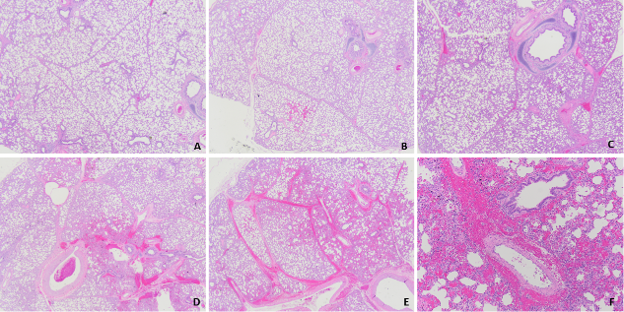

Supplement: S1 Fig — A) Score 0, where no hemorrhage is present within pulmonary parenchyma. H&E, 40X. B) Score 1, where hemorrhages affect <10% of parenchyma. H&E, 40X. C) Score 2, where hemorrhages affect >10–25% of parenchyma. H&E, 40X. D) Score 3, where hemorrhages affect >25–50% of parenchyma. H&E, 40X. E) Score 4, where hemorrhages are present >50% of parenchyma. H&E, 40X. F) The distribution of hemorrhages within the most severely affected lungs consisted of a perivascular, peribronchiolar, and interalveolar pattern. H&E, 100X. (TIF) [file pone.0328073.s001.tif]
