# Supplementary material for: Evaluation of commercially available class A water-based foam concentrates for swine depopulation
Source: PLoS One. 2025 Aug 18;20(8):e0328073. doi: 10.1371/journal.pone.0328073 (PMC12360584; doi:10.1371/journal.pone.0328073)
Supplement: S1 Table — (PDF) [file pone.0328073.s002.pdf]

S1 Table. Rubric for histological assessment of sampled organs from pigs from exposure trial experiments.

Score	Nasal Turbinate	Trachea	Lung (R Cranial)	Conjunctiva	Skin
**0**	No microscopic changes	No microscopic changes	No microscopic changes	No microscopic changes	No microscopic changes
**1**	Lamina propria inflammatory infiltration +/- epithelial deciliation	Lamina propria inflammatory infiltration +/- epithelial deciliation	Hemorrhage within <10% of parenchyma	Minimal (<10%) conjunctivitis +/- edema	Superficial dermatitis or subcorneal pustule formation
**2**	Superficial submucosal lymphoplasmacytic infiltration +/- epithelial deciliation	Superficial submucosal lymphoplasmacytic infiltration +/- epithelial deciliation	Hemorrhage within >10-25% of pulmonary parenchyma	Mild (>10-20%) conjunctivitis +/- edema	Superficial dermal edema and/or superficial dermatitis
**3**	Deep submucosal inflammatory infiltration +/- epithelial deciliation	Deep submucosal inflammatory infiltration +/- epithelial deciliation	Hemorrhage within >25-50% of pulmonary parenchyma	Moderate (>20-40%) conjunctivitis +/- edema	Widespread dermal edema and/or deep dermatitis
**4**	Full-thickness inflammatory infiltration +/- epithelial deciliation	Full-thickness inflammatory infiltration +/- epithelial deciliation	Hemorrhage within >50% of pulmonary parenchyma	Marked (>40%) conjunctivitis +/- edema	Widespread dermal edema and/or dermatitis with panniculitis
