# Supplementary material for: Evaluation of commercially available class A water-based foam concentrates for swine depopulation
Source: PLoS One. 2025 Aug 18;20(8):e0328073. doi: 10.1371/journal.pone.0328073 (PMC12360584; doi:10.1371/journal.pone.0328073)
Supplement: S2 Table — (PDF) [file pone.0328073.s003.pdf]

S2 Table. Rubric for gross pathological assessment of sampled tissues from pigs from exposure trial experiments.

Score	Skin (Inguinal region)	Skin (pinna - internal)	Cornea	Conjunctiva	Nasal mucosa	Tracheal mucosa	Lung (right cranial lobe)
**0**	No erythema or edema	No erythema or edema	No scleral injection or opacity	No hyperemia or edema	No hyperemia or edema	No hyperemia or hemorrhage	No congestion, hemorrhage, or emphysema
**1**	Mild erythema or edema	Mild erythema or edema	Mild scleral injection without opacity	Mild hyperemia or edema	Mild hyperemia or edema	Mild hyperemia and/or petechiae	Mild congestion or petechiae, no emphysema
**2**	Moderate erythema + mild edema	Moderate erythema + mild edema	Moderate scleral injection + mild opacity	Moderate hyperemia + mild edema	Moderate hyperemia + mild edema	Moderate hyperemia and/or ecchymoses	Mild to moderate congestion or ecchymoses + mild emphysema
**3**	Moderate to severe erythema + moderate edema	Moderate to severe erythema + moderate edema	Severe scleral injection + moderate opacity	Moderate to severe hyperemia + moderate edema	Moderate to severe hyperemia + moderate edema	Moderate to severe hyperemia and/or ecchymoses	Moderate congestion or ecchymoses + moderate emphysema
**4**	Severe erythema + severe edema	Severe erythema + severe edema	Severe scleral injection + severe opacity	Severe hyperemia + severe edema	Severe hyperemia + severe edema	Severe hyperemia and/or hemorrhage	Moderate to severe hyperemia or hemorrhage + severe emphysema
